# Impact of Acute Functional Decline on Frailty and Mortality in Acutely Hospitalized Older Persons

**DOI:** 10.1002/agm2.70104

**Published:** 2026-08-20

**Authors:** Ali Vahedi, Maria Eriksdotter, Hege Ihle‐Hansen, Torgeir Bruun Wyller, Anne Rita Øksengård, Brynjar Fure

**Affiliations:** ^1^ Department of Internal Medicine, Section of Geriatric Medicine Central Hospital Karlstad Region Värmland Sweden; ^2^ School of Medical Sciences Örebro University Örebro Sweden; ^3^ Division of Clinical Geriatrics, Department of Neurobiology, Care Sciences and Society Karolinska Institutet Huddinge Sweden; ^4^ Theme Inflammation and Aging Karolinska University Hospital Huddinge Sweden; ^5^ Department of Acute Medicine Oslo University Hospital Oslo Norway; ^6^ Institute of Health and Society University of Oslo Oslo Norway; ^7^ Institute of Clinical Medicine University of Oslo Oslo Norway; ^8^ Department of Geriatric Medicine Oslo University Hospital Oslo Norway; ^9^ The Norwegian Health Association Oslo Norway

**Keywords:** clinical frailty scale, cognitive domains, cognitive impairment, frailty, older persons

## Abstract

**Objectives:**

Older persons are often referred to emergency departments due to acute functional decline, that is, reduced activities of daily living occurring in the days before hospitalization, frequently driven by acute medical conditions. We aimed to describe frailty in the oldest hospitalized patients presenting with acute functional decline and evaluate predictors of death, severe frailty and nursing home admission after 3–6 months.

**Methods:**

We included 119 persons with acute functional decline, aged ≥ 80 years, ≥ 5 systemic diagnoses, ≥ 5 drugs daily, from an acute geriatric ward and reassessed after 3–6 months. All were evaluated with comprehensive geriatric assessment. Severe frailty was defined as Clinical Frailty Scale 7–8.

**Results:**

Forty‐one (34.4%) were free from severe frailty at baseline and 32 (48.5%) at follow‐up. Male sex (adjusted OR 11.820, 95% confidence interval [CI] 3.670–38.120), Clinical Dementia Rating (CDR) ≥ 0.5 (adjusted OR 3.920, 95% CI 1.020–15.100), severe frailty at baseline (adjusted OR 6.930, 95% CI 1.830–26.210), type 2 diabetes (adjusted OR 7.460, 95% CI 2.040–27.250), and cancer (adjusted OR 5.640, 95% CI 1.040–30.690) were associated with death at follow‐up. Hearing impairment (adjusted OR 7.060, 95% CI 1.430–34.760), CDR ≥ 0.5 (adjusted OR 5.390, 95% CI 1.260–23.070), and nursing home living before admission (adjusted OR 4.260, 95% CI 1.010–17.920) were associated with severe frailty at follow‐up.

**Conclusions:**

Many persons with acute functional decline have severe frailty. Male sex, cognitive impairment, diabetes mellitus type 2, cancer, hearing impairment, and nursing home living seem to increase the risk of severe frailty or death and should be considered when person‐centered care is planned for hospitalized older people.

## Graphical abstract

Older persons with acute functional decline are often severely frail. Male sex, cognitive impairment, diabetes mellitus type 2, cancer, hearing impairment, and nursing home living increase the risk of severe frailty or death and should be considered when person‐centered care is planned for hospitalized older people. CFS, Clinical Frailty Scale.

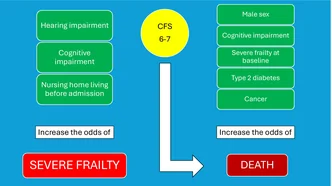

**Abbreviations**
ADL: activities of daily living
B‐ADL: basic activities of daily living
CFS: Clinical Frailty Scale
CGA: comprehensive geriatric assessment
CI: confidence interval
DOAC: direct‐acting oral anticoagulants
DSM: Diagnostic and Statistical Manual of Mental Disorders, 5th Edition
eGFR: estimated glomerular filtration rate
I‐ADL: Instrumental Activities of Daily Living
IQR: interquartile range
MCI: mild cognitive impairment
OR: odds ratio
TUG: timed up and go

## 1 Introduction

### 1.1 Acute Functional Decline

A significant decline in daily functions occurs in the days before hospitalization in 40%–65% of patients admitted to acute medical settings [1]. This impairment is often referred to as acute functional decline and leads to deficits in both basic Activities of Daily Living (B‐ADL) and instrumental ADL (I‐ADL) [1, 2]. This group mainly comprises of older persons with lower premorbid functional status and many are diagnosed with dementia [3]. While functional decline may develop during hospital stay, emerging evidence suggests that a significant part of the functional decline precedes hospitalization, with only 1%–7% of patients undergoing further functional deterioration during their stay [3].

Emergency department visits and subsequent hospitalization due to functional decline are frequently caused by acute medical conditions, and persons with acute functional decline often experience increased dependence on community support, as well as risk of transfer to a nursing home, hospital readmission and death [1, 4].

In older hospitalized persons, approximately half of new‐onset disabilities result from acute diseases, and about one‐third are discharged with worsened functional status compared with 2 weeks before admission [3]. Prehospital functional decline causes longer hospital stays and negatively impacts prognosis [3].

### 1.2 Frailty

The frailty syndrome is defined as a physiological disequilibrium leading to a reduced physiological adaptability to stressors and, subsequently, an impaired ability to return to homeostasis [5]. The syndrome presents as an increased vulnerability and may result in a sudden or gradual decrease in independence in ADL [6]. Frailty is associated with an increased risk of falls, disability, hospitalization, and mortality [7], significantly impairing the quality of life in older persons and posing a financial challenge for healthcare providers worldwide [8]. Often a subclinical condition, frailty may be overlooked as other ailments take precedence when individuals seek healthcare [9].

Many frailty screening tools have been developed and the frailty phenotype by Fried [7] and the Clinical Frailty Scale (CFS) by Rockwood [10] are among the most commonly used. However, no gold standard for identifying frailty has yet been established [11, 12].

The phenotype model, also known as the physical frailty model, is based on five phenotypic criteria: poor grip strength, reduced walking speed, exhaustion, low physical activity level, and unintentional weight loss [7]. Individuals meeting three or more criteria are classified as frail, while those meeting one or two criteria are considered prefrail, that is, they are at an increased risk of developing frailty. Individuals who do not meet any criteria are considered robust. The CFS evaluates frailty as a clinical estimate of overall fitness and ADL function on a scale of 1 through 9, where 1 indicates a fully independent and active individual, 8 a fully dependent individual who is approaching death, and 9 a terminally ill individual [6].

### 1.3 Acute Functional Decline and Frailty

Both acute functional decline and frailty are often missed in acute medical settings due to a lack of knowledge, broad definitions and misinterpretation of the clinical presentations [13, 14]. While acute functional decline describes an acute change in functional status, frailty describes a potentially progressive, chronic state of functional impairment [1, 5]. Acute functional decline is associated with frailty, and both conditions lead to increased vulnerability [3, 6] through overlapping characteristics, including reduced ADL function, disability, and multimorbidity. Early frailty screening in the emergency department may enable physicians to manage frail persons comprehensively and to plan tailored investigations and interventions [15].

Although several studies have examined acute functional decline and frailty separately, few have described the characteristics of these syndromes together. The aim of this study was to (1) describe the oldest hospitalized persons presenting with acute functional decline at the emergency department and their trajectories; and (2) study predictors for death, severe frailty, and admission to nursing homes 3–6 months after hospital discharge.

## 2 Methods

Persons of both sexes with acute functional decline who were admitted to the emergency department of Karlstad Central Hospital, Sweden, and transferred to the hospital's acute geriatric ward were consecutively included in this cohort study regardless of risk factors or disabilities and followed up after 3–6 months. All participants were aged ≥ 80 years, had ≥ 5 systemic diagnoses, and were prescribed ≥ 5 daily medications. Acute functional decline was defined as an impairment in ADL occurring up to 2 weeks prior acute admission. The only exclusion criterion was physical or mental inability to partake in diagnostic testing. Outcomes included severe frailty, nursing home admission, and death at follow‐up 3–6 months after hospital discharge. As women are reported to have more frailty than men [16], outcomes were presented by sex.

All assessments were performed by a multiprofessional team using comprehensive geriatric assessment (CGA) [17] at baseline (Day 2–6 of the hospital stay). Assessments at follow‐up were performed by a trained geriatrician.

For details regarding collected data, diagnostic instruments, and cutoffs used during the CGA assessment, see Supporting Information.

### 2.1 Statistical Analyses

Descriptive statistics were used to summarize baseline characteristics.

Power calculation was performed with G*Power 3.1.9.7 [18] and showed that a multiple regression analysis with eight independent variables would require 109 research persons.

We screened biologically plausible variables through chi‐squared tests, and if the *p* value was < 0.10, we continued analyzing predictors of severe frailty, nursing home admission and death through a logistic regression model adjusted for age, sex, and other potential explanatory variables. The Enter method was used to avoid the pitfalls of stepwise analysis [19]. Survival data are presented using life table analysis. All analyses were performed in SPSS v.28.0.1.1. In addition, trajectories are illustrated using a Sankey diagram created with Flourish Studio [20].

### 2.2 Ethical Considerations

In this vulnerable group, 76 (64%) participants were unable to provide informed consent due to cognitive impairment or disabilities such as impaired hearing and/or vision. In accordance with the decision of the Swedish Ethical Review Authority (2019‐04110), next‐of‐kin were consulted to represent the participants' wishes.

## 3 Results

### 3.1 Characteristics of Persons Admitted With Acute Functional Decline

In total, 119 persons with a mean (±SD) age of 86.7 (±4.0) years were included, of whom 58 (48.7%) were women. According to the CFS, 41 (34.4%) did not exhibit severe frailty at baseline. Baseline characteristics are presented in Table 1.

**TABLE 1:** Baseline characteristics of the study sample, *N* = 119.

	Female, *n* = 58	Male, *n* = 61	Total, *N* = 119
Age, mean (SD)	87.0 (4.3)	86.5 (3.7)	86.7 (4.0)
BMI (SD)	23.7 (5.5)	23.5 (3.8)	23.6 (4.7)
Living arrangements, *n* (%)			
Living alone			
Independent	10 (17.2)	10 (16.4)	20 (16.8)
Home care	18 (31.0)	13 (21.3)	31 (26.1)
Co‐living			
Independent	13 (22.4)	14 (23.0)	27 (22.7)
Home care	8 (13.8)	20 (32.8)	28 (23.5)
Assisted living	9 (15.5)	4 (6.6)	13 (10.9)
Risk factors, *n* (%)			
Smoking			
Current	2 (3.5)	2 (3.3)	4 (3.4)
Ex	13 (22.8)	31 (50.8)	44 (37.3)
Current alcohol consumption^a^	10 (18.2)	33 (55.0)	43 (37.4)
History of or current overconsumption^b^	1 (1.8)	6 (10.2)	7 (6.1)
Frailty phenotype, *n* (%)			
Robust	2 (3.4)	5 (8.2)	7 (5.9)
Prefrail	11 (19.0)	8 (13.1)	19 (16.0)
Frail	45 (78.2)	48 (78.7)	93 (78.2)
Clinical Frailty Scale^c^, median (IQR)	7 (1)	7 (1)	7 (1)
Clinical Frailty Scale^c^ categories, *n* (%)			
CFS 1–4	6 (10.4)	4 (6.6)	10 (8.4)
CFS 5–6	14 (24.1)	17 (27.9)	31 (26.0)
CFS 7–8	38 (65.5)	40 (65.5)	78 (65.6)
CFS 9	0 (0.0)	0 (0.0)	0 (0.0)
Comorbidities, *n* (%)			
Hypertension	44 (75.9)	42 (68.9)	86 (72.2)
Diabetes mellitus type 2	15 (25.9)	21 (34.4)	36 (30.3)
Hyperlipidemia	8 (13.8)	14 (23.0)	22 (18.5)
Heart failure	5 (8.6)	9 (14.8)	14 (11.8)
Coronary artery disease	11 (19.3)	13 (21.7)	24 (20.5)
Cancer^d^	3 (5.3)	8 (13.1)	11 (9.3)
Spread	3 (5.3)	2 (3.3)	5 (4.2)
Chronic lung disease	11 (19.0)	8 (13.1)	19 (16.0)
Medications, *n* (%)			
Neuroleptics	3 (5.2)	8 (13.1)	11 (9.2)
Benzodiazepines	9 (15.5)	3 (4.9)	12 (10.1)
SSRI/SNRI	6 (10.3)	8 (13.1)	14 (11.8)
Memantine	3 (5.2)	8 (13.3)	11 (9.3)
Platelet inhibitors^e^	18 (31.0)	14 (23.0)	32 (26.9)
Warfarin and DOAC	12 (20.7)	26 (42.6)	38 (31.9)
ACE inhibitors	22 (37.9)	23 (37.7)	45 (37.8)
Calcium inhibitors	13 (22.4)	8 (13.3)	21 (17.8)
Diuretics	14 (24.1)	30 (49.2)	44 (37.0)
Insulin	4 (6.9)	5 (8.2)	9 (7.6)
Peroral antidiabetics	7 (12.1)	9 (14.8)	16 (13.4)
Non‐peroral antidiabetics	0 (0.0)	3 (4.9)	3 (2.5)

Abbreviations: ACE = angiotensin converting enzyme, BMI = body mass index, DOAC = direct‐acting oral anticoagulants, IQR = interquartile range, SD = standard deviation, SNRI = serotonin–norepinephrine reuptake inhibitors, SSRI = selective serotonin reuptake inhibitors.

^a^ Any consumption.

^b^ Medical diagnosis of overconsumption.

^c^ There were no cases with CFS 9.

^d^ Active cancer diagnoses.

^e^ Acetylsalicylic acid or clopidogrel.

The most common tentative diagnoses assigned by the admitting physicians were delirium 33 (27.7%), infections 31 (26.1%), “causa socialis” 30 (25.2%), and heart failure 4 (3.4%). Other causes 21 (17.6%) included respiratory failure, for example, exacerbation of chronic obstructive pulmonary disease or asthma, and prerenal kidney failure, stroke, falls, or unspecified preliminary diagnoses.

At discharge, that is, after CGA and other relevant diagnostic investigations, diagnoses were revised. The largest group of main diagnoses comprised cognitive impairments, including MCI 41 (34.5%), dementia 56 (47.1%), and delirium independently 20 (43.7%), or as part of delirium superimposed on dementia 21 (17.6%). Infections were diagnosed in 37 (31.1%), heart failure in 15 (12.6%), and other diagnoses, comprising respiratory failure, prerenal kidney failure, electrolyte imbalances, cancer, falls, and stroke, in 37 (31.1%). In many cases, these diagnoses overlapped.

Many persons fulfilled the diagnostic criteria for dementia at baseline after CGA, 56 (47.0%), followed by MCI 41 (34.5%) and no cognitive impairment, 22 (18.5%). Dementia etiologies were mixed dementia etiology (Alzheimer's and vascular disease) in 36 (30.3%), Alzheimer's dementia in 15 (12.6%), vascular dementia in 4 (3.4%), and Parkinson's dementia in 1 (0.8%).

Persons admitted under the label “causa socialis” were subsequently diagnosed with cognitive impairments due to MCI in 14 (33.3%), dementia 22 (52.4%), or delirium either independently in 8 (35.7%) or as part of delirium superimposed on dementia in 7 (16.7%), followed by infections in 10 (23.8%), heart failure in 3 (7.1%), and other conditions 10 in (21.4%).

### 3.2 Survival, Frailty Status, and Living Arrangements at Follow‐Up

A greater proportion of women than men remained alive at follow‐up, 49 (87.5%) versus 30 (49.2%).

Among the 79 participants surviving until follow‐up, 18 (22.8%) were unable to participate in a physical follow‐up. The CFS score was unchanged in 33 (41.8%), worsened in 16 (20.2%), and improved in 12 (15.2%).

**FIGURE 1:**
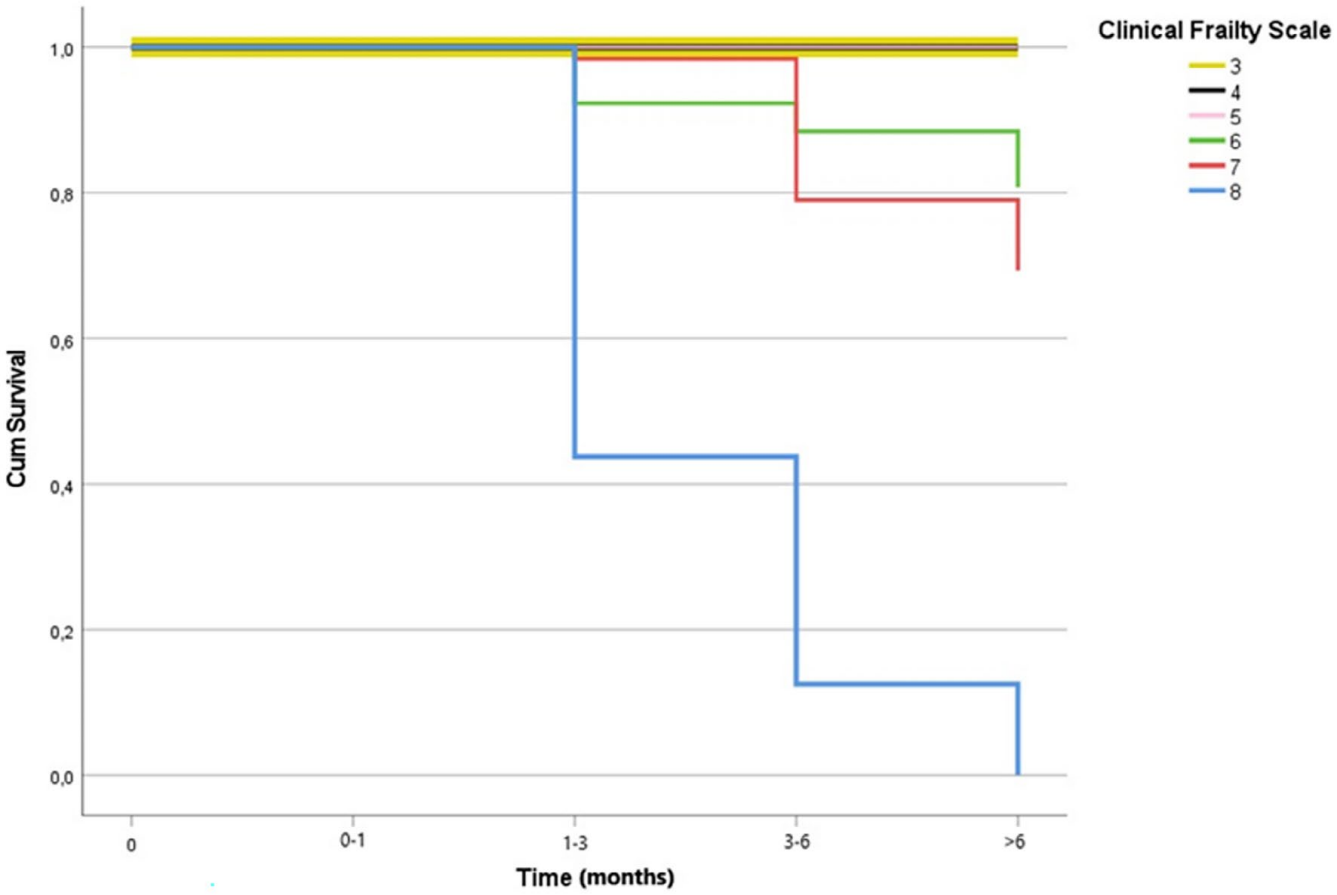
Survival stratified by Clinical Frailty Scale category.

No persons were assessed as CFS 1 or 2. Mortality increased with higher frailty status. All individuals with CFS 3–5 were alive at 6 months, compared with 80.8% of those with CFS 6, 69.4% of CFS 7, and 0% of CFS 8 (see Figure 1).

**FIGURE 2:**
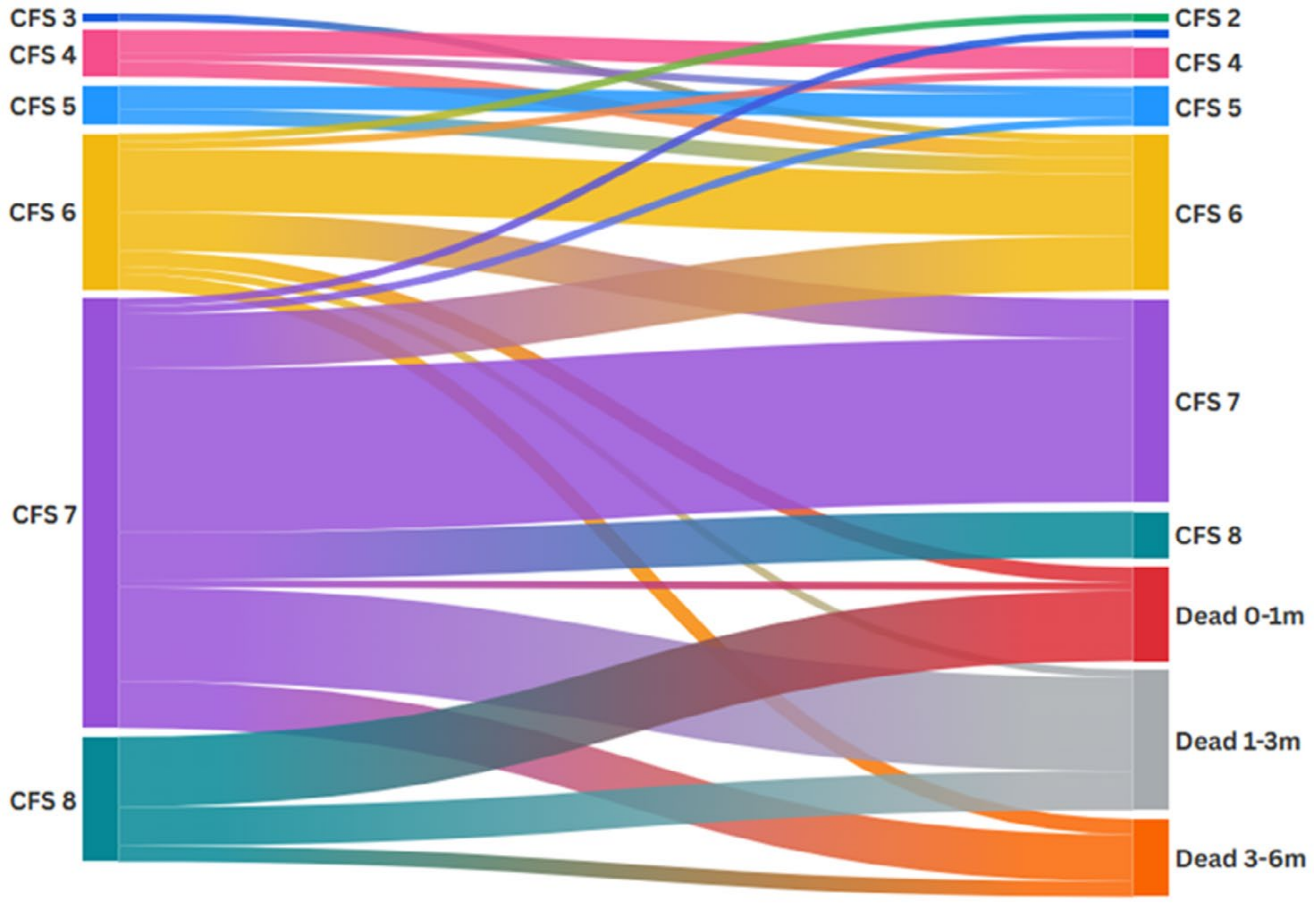
Trajectory analysis showing Clinical Frailty Scale and death at follow‐up. No persons were assessed as CFS 1 and 2. CFS = Clinical Frailty Scale, m = month(s).

Trajectories were analyzed with respect to CFS and survival (see Figure 2). Persons with CFS 3–5 were relatively stable, with few progressing in frailty status, whereas those with more severe frailty progressed in frailty status or died.

At 3‐ to 6‐month follow‐up, slightly more women lived alone with home care than men, 4 (19.0%) versus 4 (16.7%) and co‐lived with support from home care, 8 (38.1%) versus 8 (33.3%). More men lived with assisted living compared to women, 9 (37.5%) versus 6 (28.6%). Overall, more persons lived with home care and assistance at 3‐ to 6‐month follow‐up compared to baseline.

### 3.3 Comparison of the Follow‐Up and Lost‐to‐Follow‐Up Groups

Comparing the lost‐to‐follow‐up group (*n* = 18) with the follow‐up group (*n* = 61), the age distribution was similar. Both groups had more women than men. More participants lived alone with home care in the follow‐up group, and in the lost‐to‐follow‐up group, more persons co‐lived with no assistance or lived in nursing homes. Physical frailty was more prevalent in the lost‐to‐follow‐up group, 16 (88.8%) versus 40 (65.6%). According to the CFS, frailty status was similar between the groups (see Table 2).

**TABLE 2:** Comparison between the follow‐up group and the lost‐to‐follow‐up group.

	Follow‐up, *n* = 61	Lost‐to‐follow‐up, *n* = 18	*p*
Age, mean (SD)	86.7 (4.18)	87.2 (3.86)	< 0.001
BMI (SD)	24.2 (4.91)	24.0 (5.26)	< 0.001
Sex, *n* (%)			
Female	38 (62.3)	11 (61.1)	< 0.001
Male	23 (37.7)	7 (38.9)	< 0.001
Living arrangements, *n* (%)			
Living alone			
Independent	12 (19.7)	2 (11.1)	< 0.001
Home care	21 (34.4)	3 (16.7)	< 0.001
Co‐living			
Independent	10 (16.4)	8 (44.4)	< 0.001
Home care	13 (21.3)	3 (16.7)	< 0.001
Assisted living	5 (8.2)	2 (11.1)	< 0.001
Risk factors, *n* (%)			
Smoking, current and ex	21 (34.4)	7 (38.9)	< 0.001
Current alcohol consumption^a^	20 (32.8)	4 (22.2)	< 0.001
Frailty phenotype, *n* (%)			
Robust	6 (9.8)	1 (5.6)	< 0.001
Prefrail	15 (24.6)	1 (5.6)	< 0.001
Frail	40 (65.6)	16 (88.8)	< 0.001
Clinical Frailty Scale^b^ categories, *n* (%)			
CFS 1–4	8 (13.1)	2 (11.1)	< 0.001
CFS 5–6	20 (32.8)	6 (33.3)	< 0.001
CFS 7–8	33 (54.1)	10 (55.1)	< 0.001
CFS 9	0 (0.0)	0 (0.0)	< 0.001
Comorbidities, *n* (%)			
Hypertension	44 (72.1)	14 (77.8)	< 0.001
Diabetes mellitus type 2	18 (29.5)	12 (66.7)	< 0.001
Hyperlipidemia	12 (19.7)	6 (33.3)	< 0.001
Coronary artery disease	11 (18.0)	6 (33.3)	
Cancer^c^	3 (4.9)	0 (0.0)	< 0.001
Spread	1 (1.6)	0 (0.0)	< 0.001
Chronic lung disease	9 (14.8)	3 (16.7)	< 0.001
Medications, *n* (%)			
Neuroleptics	3 (4.9)	2 (11.1)	< 0.001
Benzodiazepines	6 (9.8)	2 (11.1)	< 0.001
SSRI/SNRI	7 (11.4)	1 (5.6)	< 0.001
Memantine	6 (9.8)	2 (11.1)	< 0.001
Platelet inhibitors^d^	17 (27.9)	7 (38.9)	< 0.001
Warfarin and DOAC	16 (26.2)	6 (33.3)	< 0.001
ACE inhibitors	21 (34.4)	9 (50.0)	< 0.001
Calcium inhibitors	12 (19.7)	3 (16.7)	< 0.001
Diuretics	19 (31.1)	5 (27.7)	< 0.001
Insulin	5 (8.2)	3 (16.7)	< 0.001
Peroral antidiabetics	7 (11.5)	5 (27.8)	< 0.001
Non‐peroral antidiabetics	3 (4.9)	0 (0.0)	< 0.001

Abbreviations: ACE = angiotensin converting enzyme, BMI = body mass index, DOAC = direct‐acting oral anticoagulants, SD = standard deviation, SNRI = serotonin–norepinephrine reuptake inhibitors, SSRI = selective serotonin reuptake inhibitors.

^a^ Any consumption.

^b^ There were no cases with CFS 9.

^c^ Active cancer diagnoses.

^d^ Acetylsalicylic acid or clopidogrel.

Slightly more persons had a current or previous history of smoking in the lost‐to‐follow‐up group, and more participants consumed alcohol in the follow‐up group. BMI was similar between the groups. Comparing diagnoses, more persons in the lost‐to‐follow‐up group had hypertension, diabetes type 2, hyperlipidemia, and coronary artery disease (see Table 2).

### 3.4 Predictors of Severe Frailty, Death, and Nursing Home Admission

Hearing impairment, a Clinical Dementia Rating score ≥ 0.5 and nursing home living status at admission were associated with increased odds of severe frailty (CSF 7–8) status at 3–6 months follow‐up (see Table 3).

**TABLE 3:** Predictors of severe frailty and death at follow‐up.

Variable	Unadjusted OR (95% CI)	Adjusted OR^b^ (95% CI)
Severe frailty		
Age	2.060 (0.460–9.260)	2.340 (0.310–17.840)
Male gender	0.620 (0.220–1.760)	0.560 (0.120–2.540)
Hearing loss	**6.530** (1.390–30.710)	**7.060** (1.430–34.760)
Living status at admission^c^	**4.000** (1.000–15.960)	**4.260** (1.010–17.920)
Clinical Dementia Rating score ≥ 0.5	**5.710** (1.360–24.060)	**5.390** (1.260–23.070)
Death		
Age	0.740 (0.220–2.470)	1.150 (0.280–4.840)
Male gender	5.660 (2.370–13.540)	**11.820** (3.670–38.120)
Clinical Dementia Rating score ≥ 0.5	2.340 (0.730–7.480)	**3.920** (1.020–15.100)
Severe frailty^a^	**5.220** (1.600–17.060)	**6.930** (1.830–26.210)
Diabetes mellitus type 2	**4.250** (1.410–12.820)	**7.460** (2.040–27.250)
Cancer	**6.160** (1.360–27.870)	**5.640** (1.040–30.690)

*Note:* Statistically significant values in bold, *α*‐level < 0.05.

Abbreviations: CI = confidence interval, OR = odds ratio.

^a^ Defined as CFS 7–8 at baseline.

^b^ Adjusted for age and sex.

^c^ Living compared to not living in a nursing home.

Male sex, Clinical Dementia Rating score ≥ 0.5, severe frailty, diabetes mellitus type 2, and cancer were associated with increased odds of death at 3–6 months follow‐up.

Chi‐square analyses were performed to examine factors associated with transfer to a nursing home, including age, CFS, Katz‐ADL, Clinical Dementia Rating score, and eGFR. No statistically significant variables were identified for inclusion into the model [21].

## 4 Discussion

We found that participants presenting to the emergency department with acute functional decline were often diagnosed with severe frailty. In addition, admission to an acute geriatric unit was frequently motivated by cognitive impairment, often accompanied by delirium, precipitated by infection or other acute medical conditions. Hearing impairment, nursing home living, and a Clinical Dementia Rating score ≥ 0.5 at baseline were statistically significant predictors of severe frailty status at follow‐up. Male sex, a Clinical Dementia Rating score ≥ 0.5, severe frailty, diabetes mellitus type 2, and cancer were associated with a statistically significant increase in the odds of death at follow‐up.

In this study, undiagnosed cognitive impairment was highly prevalent in persons admitted with acute functional decline and both cognitive function and delirium should therefore be routinely assessed with delirium screening, alongside other conditions in the acute medical setting. Older age and cognitive impairments are significant predictors of acute functional decline, whereas a higher premorbid function is protective [3]. Acute disease is thought to impact the functional status of vulnerable persons, leading to a chronic functional decline that may in turn be a predictor of frailty [3]. It is crucial that the clinician identifies and treats the causes of acute functional decline, as it is not a part of physiological aging.

Functional limitations are highly associated with frailty, and although these concepts are considered distinct, acute functional decline, and frailty share many common developmental pathways [22]. Methods for identifying persons at risk for acute functional decline include screening for shared risk factors, such as biological age, cognitive impairment, delirium, previous hospitalizations, and lack of social support [23, 24, 25].

Most frailty screening tools include a measure of functional impairment, which may be considered an integral part of the frailty syndrome [10, 26]. Therefore, we propose that acute functional decline should be explored as a possible manifestation of underlying frailty. Clinicians should regularly screen for frailty in all healthcare settings to guide risk assessment, establish treatment plans including direction of care for older persons and interventions to halt the progress of frailty [25, 27, 28, 29].

Hearing impairment, nursing home living and cognitive impairment increased the odds of severe frailty, as supported by previous studies [25]. Impaired hearing has been reported to increase the risk of falls, poor physical function and reduced activities of daily living, sharing pathways with frailty and cognitive impairment [30]. However, it remains unknown whether this association is causal. While several studies suggest that the use of hearing aids could potentially alleviate frailty and cognitive impairment, systematic reviews have found the evidence to be inconclusive [30, 31].

Nursing home living is an indicator of low ADL function and frailty, and in Swedish settings, limited B‐ADL function is generally required for admission to nursing homes [32, 33]. Our negative results regarding predictors of nursing home living after 3–6 months, could be explained by the short follow‐up period and the high proportion of participants with severe frailty at baseline. Additionally, in Sweden, citizens are encouraged to “age in place”, that is, live in ordinary housing with home visits from nursing staff providing home healthcare or subsidized home‐based care for support with ADL [34].

The increased odds of mortality of male sex may reflect the well‐documented frailty paradox, which describes the biological phenomenon whereby women live longer than men despite higher levels of comorbidity and functional impairment [16]. It is hypothesized that women have a higher physiological reserve and accumulate more deficits that negatively impact function, quality of life, and self‐reported health over time, whereas men take more risks, adhere less consistently to prescribed preventative medications, and have a higher prevalence of lethal comorbidities [16].

Chronological age was not a predictor of adverse outcomes in this study. In contrast, frailty is a strong predictor of increased all‐cause mortality. The present cohort consisted of individuals of advanced chronological age, supporting that frailty status, which reflects the individual's biological age, is more important than chronological age in predicting mortality even among the oldest adults. A systematic review and meta‐analysis concluded that frailty and all‐cause mortality are significantly associated in persons under 90 years of age, although this association could not be confirmed in those over 90 years of age [35].

Severe dementia, as indicated by a high Clinical Dementia Rating score, increased the odds of death [36, 37]. As expected, dementia severity is associated with higher rates of physical impairment, undernutrition, and increased mortality [36].

Persons with diabetes mellitus type 2 were also at increased risk of death, and previous studies have indicated that intensive glycemic control increases mortality risk and fails to reduce the risk of developing cardiovascular disease and end stage renal disease in persons 80 years or older [38]. In this population, treatment of diabetes type 2 should focus on preventing hypoglycemia rather than tightly regulating glycemic control.

Cancer incidence has increased globally, with 50% of cases occurring in individuals aged 65 years or older, even though this age group comprises only 8% of the global population, particularly in the oldest‐old [39]. Additionally, cancer is often diagnosed at later stages in this group, and mortality remains high even for cancer diagnoses with relatively favorable prognoses.

Emergency physicians focus on rapid and accurate organ‐specific diagnoses [40], and it is well recognized that “diffuse” states, such as acute functional decline, severe frailty and cognitive impairment, are labeled as “failure to cope,” “weak and dizzy” [41], and in Nordic countries, “causa socialis.” To our knowledge, “causa socialis” best describes a nonmedical situation where a person is admitted to a hospital due to lacking social resources. The term is not an established diagnosis in ICD‐10 [42], and should generally be avoided. No persons in the present sample were admitted solely due to lacking social support, suggesting that the term mislabels older frail persons admitted to geriatric wards.

### 4.1 Strengths and Limitations

This study had some limitations. Due to advanced age and disabilities, many persons were unable to attend the follow‐up. We had to extend follow‐up time from 3 to 6 months to allow individuals to participate in the follow‐up visits. The follow‐up cohort consisted of the least frail persons in the sample, introducing selection bias and potentially leading to an overestimation of the associations with physical frailty. Additionally, we could not assess frailty prior to hospital admission, as frailty is not routinely evaluated at most outpatient clinics in the region, and patients, next‐of‐kin or nursing staff could not provide reliable information on functional status before the hospital stay. The inclusion process was affected by known barriers in geriatric research, such as complex diseases and impaired ADL function, vision, hearing, and capacity to provide informed consent [43, 44]. Consequently, this study was small, including only 119 research persons, and the results must be interpreted with caution.

Strengths of the study included the inclusion of research persons typically excluded from research, such as very old individuals with cognitive impairment, visual or hearing impairment, and low ADL function [45, 46]. This reduces selection bias and improves the generalizability of the results [47]. We also employed validated diagnostic instruments in the CGA, administered by a trained multiprofessional team.

## 5 Conclusion

We found that 35% of hospitalized persons presenting to the emergency department with acute functional decline did not meet the criteria for severe frailty. Older persons admitted to acute geriatric units with acute functional decline frequently exhibit cognitive impairment, with or without delirium, which should be assessed routinely in the acute medical setting using validated brief screening instruments. Persons with hearing impairment, those living in nursing homes, and those with cognitive impairment may be at increased risk of severe frailty. We propose that hearing loss, as a modifiable risk factor, should be routinely screened for and managed with appropriate hearing aids. Male sex, cognitive impairment, severe frailty, diabetes mellitus type 2, and cancer may increase the risk of death and should be considered when planning individualized diagnostic procedures and treatments. Further studies are needed to explore the relationship between acute functional decline and frailty in the oldest adults.

## Author Contributions

A.V. and B.F. developed the idea behind the study and set the PICO. A.V. extracted the data, and A.V. and B.F. performed the analyses. T.B.W., H.I.‐H., M.E., and A.R.Ø. reviewed the project plan, read and revised the manuscript, and provided inputs regarding the interpretation of results and discussion of the findings.

## Funding

The study was founded by the School of Medical Sciences, Örebro University, Örebro, Sweden, and Karlstad Central Hospital, Region Värmland, Sweden.

## Ethics Statement

This study was conducted in accordance with the World Medical Associations Declaration of Helsinki. Ethical approval was gained from the Swedish Ethical Review Authority before the commencement of the inclusion process (Dnr: 2020‐01627).

## Consent

Each participant gave informed consent, and if unable to, next‐of‐kin was consulted to represent his/her wishes.

## Conflicts of Interest

The authors declare no conflicts of interest.

## Supporting information


**Appendix S1:** Data collection and diagnostic instruments.

## Data Availability

The data that support the findings of this study are available from the corresponding author upon reasonable request.

## References

[1] O. Bogler , D. Kirkwood , P. C. Austin , et al., “Recent Functional Decline and Outpatient Follow‐Up After Hospital Discharge: A Cohort Study,” BMC Geriatrics 23, no. 1 (2023): 550, 10.1186/s12877-023-04192-7. PMC10496187 37697250

[2] A. Palese , S. Gonella , R. Moreale , et al., “Hospital‐Acquired Functional Decline in Older Patients Cared for in Acute Medical Wards and Predictors: Findings From a Multicentre Longitudinal Study,” Geriatric Nursing 37, no. 3 (2016): 192–199, 10.1016/j.gerinurse.2016.01.001. 26895646

[3] L. Palleschi , F. L. Fimognari , A. Pierantozzi , et al., “Acute Functional Decline Before Hospitalization in Older Patients,” Geriatrics & Gerontology International 14, no. 4 (2014): 769–777, 10.1111/ggi.12160. 24112396

[4] L. Geyskens , A. Jeuris , M. Deschodt , B. Van Grootven , E. Gielen , and J. Flamaing , “Patient‐Related Risk Factors for In‐Hospital Functional Decline in Older Adults: A Systematic Review and Meta‐Analysis,” Age and Ageing 51, no. 2 (2022): afac007, 10.1093/ageing/afac007. 35165688

[5] D. H. Kim and K. Rockwood , “Frailty in Older Adults,” New England Journal of Medicine 391, no. 6 (2024): 538–548, 10.1056/NEJMra2301292. PMC11634188 39115063

[6] L. Fratiglioni , L. J. Launer , K. Andersen , et al., “Incidence of Dementia and Major Subtypes in Europe: A Collaborative Study of Population‐Based Cohorts. Neurologic Diseases in the Elderly Research Group,” Neurology 54, no. 11 S5 (2000): S10–S15. 10854355

[7] L. P. Fried , C. M. Tangen , J. Walston , et al., “Frailty in Older Adults: Evidence for a Phenotype,” Journals of Gerontology Series A: Biological Sciences and Medical Sciences 56, no. 3 (2001): M146–M156, 10.1093/gerona/56.3.m146. 11253156

[8] A. M. Negm , C. C. Kennedy , L. Thabane , et al., “Management of Frailty: A Protocol of a Network Meta‐Analysis of Randomized Controlled Trials,” Systematic Reviews 6, no. 1 (2017): 130, 10.1186/s13643-017-0522-7. PMC5499023 28679416

[9] J. Walston , E. C. Hadley , L. Ferrucci , et al., “Research Agenda for Frailty in Older Adults: Toward a Better Understanding of Physiology and Etiology: Summary From the American Geriatrics Society/National Institute on Aging Research Conference on Frailty in Older Adults,” Journal of the American Geriatrics Society 54, no. 6 (2006): 991–1001, 10.1111/j.1532-5415.2006.00745.x. 16776798

[10] K. Rockwood , X. Song , C. MacKnight , et al., “A Global Clinical Measure of Fitness and Frailty in Elderly People,” CMAJ: Canadian Medical Association Journal 173, no. 5 (2005): 489–495, 10.1503/cmaj.050051. PMC1188185 16129869

[11] E. Dolenc and D. Rotar‐Pavlič , “Frailty Assessment Scales for the Elderly and Their Application in Primary Care: A Systematic Literature Review,” Zdrastveno Varstvo 58, no. 2 (2019): 91–100, 10.2478/sjph-2019-0012. PMC6455011 30984300

[12] H. Lee , E. Lee , and I. Y. Jang , “Frailty and Comprehensive Geriatric Assessment,” Journal of Korean Medical Science 35, no. 3 (2020): e16, 10.3346/jkms.2020.35.e16. PMC6970074 31950775

[13] L. Lee , G. Heckman , and F. J. Molnar , “Frailty: Identifying Elderly Patients at High Risk of Poor Outcomes,” Canadian Family Physician 61, no. 3 (2015): 227–231. PMC4369632 25767167

[14] K. Beaton , C. McEvoy , and K. Grimmer , “Identifying Indicators of Early Functional Decline in Community‐Dwelling Older People: A Review,” Geriatrics & Gerontology International 15, no. 2 (2015): 133–140, 10.1111/ggi.12379. 25303103

[15] J. D. van Oppen and M. Suzanne , “Frailty Screening in the Emergency Department: Why Does It Matter?,” Age and Ageing 53, no. 4 (2024): afae056, 10.1093/ageing/afae056. 38655587

[16] E. H. Gordon , N. M. Peel , M. Samanta , O. Theou , S. E. Howlett , and R. E. Hubbard , “Sex Differences in Frailty: A Systematic Review and Meta‐Analysis,” Experimental Gerontology 89 (2017): 30–40, 10.1016/j.exger.2016.12.021. 28043934

[17] G. Ellis , M. Gardner , A. Tsiachristas , et al., “Comprehensive Geriatric Assessment for Older Adults Admitted to Hospital,” Cochrane Database of Systematic Reviews 9, no. 9 (2017): CD006211, 10.1002/14651858.CD006211.pub3. PMC6484374 28898390

[18] F. Faul , E. Erdfelder , A.‐G. Lang , and A. Buchner , “G*Power 3: A Flexible Statistical Power Analysis Program for the Social, Behavioral, and Biomedical Sciences,” Behavior Research Methods 39, no. 2 (2007): 175–191, 10.3758/BF03193146. 17695343

[19] C. Judd , G. McClelland , and C. Ryan , Data Analysis: A Model Comparison Approach, 2nd ed. (Routledge, 2009).

[20] Flourish , “Flourish Studio” [Online] (2025), https://flourish.studio/.

[21] B. Brorsson and K. H. Asberg , “Katz Index of Independence in ADL. Reliability and Validity in Short‐Term Care,” Scandinavian Journal of Rehabilitation Medicine 16, no. 3 (1984): 125–132. 6494836

[22] R. Milte and M. Crotty , “Musculoskeletal Health, Frailty and Functional Decline,” Best Practice & Research. Clinical Rheumatology 28, no. 3 (2014): 395–410, 10.1016/j.berh.2014.07.005. 25481423

[23] J. P. A. Tavares , L. Nunes , and J. C. G. Grácio , “Hospitalized Older Adult: Predictors of Functional Decline,” Revista Latino‐Americana de Enfermagem 29 (2021): e3399, 10.1590/1518-8345.3612.3399. PMC7798389 33439951

[24] X. Wang , J. Hu , and D. Wu , “Risk Factors for Frailty in Older Adults,” Medicine 101, no. 34 (2022): e30169. 10.1097/MD.0000000000030169 PMC9410572 36042657

[25] E. Dent , P. Hanlon , M. Sim , et al., “Recent Developments in Frailty Identification, Management, Risk Factors and Prevention: A Narrative Review of Leading Journals in Geriatrics and Gerontology,” Ageing Research Reviews 91 (2023): 102082, 10.1016/j.arr.2023.102082. 37797723

[26] S. A. Sternberg , A. W. Schwartz , S. Karunananthan , H. Bergman , and A. M. Clarfield , “The Identification of Frailty: A Systematic Literature Review,” Journal of the American Geriatrics Society 59, no. 11 (2011): 2129–2138, 10.1111/j.1532-5415.2011.03597.x. 22091630

[27] E. O. Hoogendijk , J. Afilalo , K. E. Ensrud , P. Kowal , G. Onder , and L. P. Fried , “Frailty: Implications for Clinical Practice and Public Health,” Lancet 394, no. 10206 (2019): 1365–1375, 10.1016/S0140-6736(19)31786-6. 31609228

[28] P. Mehta , G. Lemon , L. Hight , et al., “A Systematic Review of Clinical Practice Guidelines for Identification and Management of Frailty,” Journal of Nutrition, Health and Aging 25, no. 3 (2021): 382–391, 10.1007/s12603-020-1549-3. PMC12280659 33575732

[29] S. Juma , M. M. Taabazuing , and M. Montero‐Odasso , “Clinical Frailty Scale in an Acute Medicine Unit: A Simple Tool That Predicts Length of Stay,” Canadian Geriatrics Journal 19, no. 2 (2016): 34–39, 10.5770/cgj.19.196. PMC4922366 27403211

[30] R. Tian , O. P. Almeida , D. M. P. Jayakody , and A. H. Ford , “Association Between Hearing Loss and Frailty: A Systematic Review and Meta‐Analysis,” BMC Geriatrics 21, no. 1 (2021): 333, 10.1186/s12877-021-02274-y. PMC8147347 34034656

[31] M. E. Sanders , E. Kant , A. L. Smit , and I. Stegeman , “The Effect of Hearing Aids on Cognitive Function: A Systematic Review,” PLoS One 16, no. 12 (2021): e0261207, 10.1371/journal.pone.0261207. PMC8719768 34972121

[32] M. Zhao , Z. Yang , Y. Wang , M. Li , and K. Wang , “Resident‐ and Institutional‐Level Factors, Frailty, and Nursing Homes Residents,” Nursing Research 71, no. 1 (2022): E1–E9, 10.1097/nnr.0000000000000556. 34620773

[33] A. S. Källberg , L. M. Berg , S. Skogli , C. Bjurbo , Å. Muntlin , and A. Ehrenberg , “Prevalence of Frailty and Associated Factors in Older Adults Seeking Care at Swedish Emergency Departments,” BMC Geriatrics 23, no. 1 (2023): 798, 10.1186/s12877-023-04545-2. PMC10694934 38049748

[34] L. Spang , M. Holmefur , L. Hermansson , and K. Lidström Holmqvist , “Applying to a Nursing Home Is a Way to Maintain Control of Life‐Experiences From Swedish Nursing Home Applicants,” Scandinavian Journal of Caring Sciences 37, no. 1 (2023): 106–116, 10.1111/scs.13104. 35778880

[35] J. A. Tan , J. H. Koh , R. A. Merchant , and L. F. Tan , “Frailty as a Predictor of Mortality in the Oldest Old: A Systematic Review and Meta‐Analysis,” Geriatrics & Gerontology International 25, no. 1 (2025): 102–107, 10.1111/ggi.15025. 39581630

[36] R. D. Piovezan , D. Oliveira , N. Arias , D. Acosta , M. J. Prince , and C. P. Ferri , “Mortality Rates and Mortality Risk Factors in Older Adults With Dementia From Low‐ and Middle‐Income Countries: The 10/66 Dementia Research Group Population‐Based Cohort Study,” Journal of Alzheimer's Disease 75, no. 2 (2020): 581–593, 10.3233/jad-200078. PMC7306886 32310178

[37] A. Sanders , “Clinical Dementia Rating Scores and Prediction of Mortality in Older Adults (P01.084),” Neurology 78, no. 1S (2012): P01.084, 10.1212/WNL.78.1_supplement.P01.084.

[38] E. S. Huang , N. Laiteerapong , J. Y. Liu , P. M. John , H. H. Moffet , and A. J. Karter , “Rates of Complications and Mortality in Older Patients With Diabetes Mellitus: The Diabetes and Aging Study,” JAMA Internal Medicine 174, no. 2 (2014): 251–258, 10.1001/jamainternmed.2013.12956. PMC3950338 24322595

[39] S. Pilleron and E. Bastiaannet , “Epidemiology of Cancer in Older Adults: A Systematic Review,” Current Oncology Reports 26, no. 9 (2024): 1021–1046, 10.1007/s11912-024-01567-w. 38963522

[40] J. Niederdöckl , N. Buchtele , M. Schwameis , and H. Domanovits , “Modern Diagnostics in Emergency Medicine,” Wiener Klinische Wochenschrift 133, no. 5–6 (2021): 249–266, 10.1007/s00508-020-01657-2. PMC7204114 32356102

[41] “Acute Functional Decline,” Geri‐EM, accessed February 11, 2025.

[42] World Health Organization , “International Statistical Classification of Diseases and Related Health Problems, 10th Revision” 6th ed.

[43] L. Mody , D. K. Miller , J. M. McGloin , et al., “Recruitment and Retention of Older Adults in Aging Research,” Journal of the American Geriatrics Society 56, no. 12 (2008): 2340–2348, 10.1111/j.1532-5415.2008.02015.x. PMC2695395 19093934

[44] S. Ross , A. Grant , C. Counsell , W. Gillespie , I. Russell , and R. Prescott , “Barriers to Participation in Randomised Controlled Trials: A Systematic Review,” Journal of Clinical Epidemiology 52, no. 12 (1999): 1143–1156, 10.1016/s0895-4356(99)00141-9. 10580777

[45] D. Rios , S. Magasi , C. Novak , and M. Harniss , “Conducting Accessible Research: Including People With Disabilities in Public Health, Epidemiological, and Outcomes Studies,” American Journal of Public Health 106, no. 12 (2016): 2137–2144, 10.2105/ajph.2016.303448. PMC5104996 27736212

[46] M. Thake and A. Lowry , “A Systematic Review of Trends in the Selective Exclusion of Older Participant From Randomised Clinical Trials,” Archives of Gerontology and Geriatrics 72 (2017): 99–102, 10.1016/j.archger.2017.05.017. 28618323

[47] J. Ruske , G. Sharma , K. Makie , et al., “Patient Comprehension Necessary for Informed Consent for Vascular Procedures Is Poor and Related to Frailty,” Journal of Vascular Surgery 73, no. 4 (2021): 1422–1428, 10.1016/j.jvs.2020.06.131. 32835789

