# Supplementary material for: Impact of Acute Functional Decline on Frailty and Mortality in Acutely Hospitalized Older Persons
Source: Aging Med (Milton). 2026 Aug 20;9(4):374–83. doi: 10.1002/agm2.70104 (PMC13491115; doi:10.1002/agm2.70104)
Supplement: Supplementary file 1 [file AGM2-9-374-s001.docx]

**Supplementary material**

Data collection

Data were collected regarding suspected diagnosis at admission, age, sex, living arrangements, smoking and alcohol use from the patients’ medical records. In addition, we collected data regarding vascular risk factors including weight, height, body mass index (BMI), known diagnoses of hypertension, hyperlipidaemia and diabetes, other medical diagnoses including chronic heart and lung disease and active cancer diagnoses, as well as current medications. The final diagnoses at discharge were based on investigations made during the hospital stay.

Diagnostic instruments and cutoffs used during Comprehensive Geriatric Assessment

ADL was assessed by the Katz ADL index [1].

Hand grip strength was measured with a JAMAR dynamometer [2], gait through Timed Up and Go (TUG) [3], physical activity levels and fatigue through a self-reported assessment, and involuntary weight loss through patient information and/or existing measurements in the patient’s medical records. Information was collected from next-of-kin, home help service staff or nursing staff regarding physical activity levels and involuntary weight loss when the research person could not provide a reliable statement due to cognitive impairment or persisting delirium.

Delirium screening was performed through the 4AT test [4].

The Mini Nutritional Assessment (MNA) [5] was used to evaluate nutritional state.

The Comprehensive Geriatric Assessment (CGA) was used to classify frailty through the frailty phenotype (7) and Clinical Frailty Scale (11).

After CGA, both previously known diagnoses and new cases of mild cognitive impairment (MCI) and dementia were assessed according to the DSM-5 [6] criteria including proposed underlying pathologies. While the DSM-5 denominates MCI and dementia as minor or major neurocognitive disorders, we opted to use the terms MCI and dementia due to the common use of the terms in society, among patients and in medical literature [7]. All pre-existing cognitive diagnoses were re-assessed according to the DSM-5 criteria. We categorized level of cognitive decline according to the Clinical Dementia Rating scale.

CFS is often collapsed into three categories of frailty, CFS 1–4 non-frail, 5–6 mild-to-moderately frail and 7–8 severely frail [8]. We did not include CFS 9 in the analyses as this is defined as the presence of a terminal disease in non-frail persons. Considering the low prevalence of robust persons in this sample, we dichotomized frailty into a CFS of 1-6 describing robust to moderately frail patients and 7-8 describing those who were severely frail. Frailty phenotype was categorized according to Fried’s criteria [9]. We chose to include both frailty phenotype and CFS at baseline, since frailty phenotype focuses on physical criteria while CFS is a measure of global functioning.

Clinical Dementia Rating scores <0.5 consist of symptoms that are part of the physiological cognitive ageing process [10], and are by definition classified as normal, while scores of 0.5 or higher were deemed pathological. Clinical Dementia Rating measures cognitive impairment through dementia severity ranging from mild cognitive impairment (0.5) to severe dementia (3).

The 4AT was categorized as no delirium (0-3 points) or possible delirium (≥4 points). Chronic kidney disease (CKD) is defined as abnormalities of kidney structure and function or glomerular filtration rate (GFR) <60 mL/min/1.73 m^2^ and was grouped accordingly into CKD or no CKD [11]. Some variables were dichotomized due to a significantly skewed distribution [12].

References:

[1]	B. Brorsson and K. H. Asberg, "Katz index of independence in ADL. Reliability and validity in short-term care," (in eng), *Scand J Rehabil Med,* vol. 16, no. 3, pp. 125-32, 1984.

[2]	G. F. Hamilton, C. McDonald, and T. C. Chenier, "Measurement of grip strength: validity and reliability of the sphygmomanometer and jamar grip dynamometer," (in eng), *J Orthop Sports Phys Ther,* vol. 16, no. 5, pp. 215-9, 1992, doi: 10.2519/jospt.1992.16.5.215.

[3]	C. J. Nightingale, S. N. Mitchell, and S. A. Butterfield, "Validation of the Timed Up and Go Test for Assessing Balance Variables in Adults Aged 65 and Older," (in eng), *J Aging Phys Act,* vol. 27, no. 2, pp. 230-233, Apr 1 2019, doi: 10.1123/japa.2018-0049.

[4]	G. Bellelli *et al.*, "Validation of the 4AT, a new instrument for rapid delirium screening: a study in 234 hospitalised older people," (in eng), *Age Ageing,* vol. 43, no. 4, pp. 496-502, Jul 2014, doi: 10.1093/ageing/afu021.

[5]	E. Reber, F. Gomes, M. F. Vasiloglou, P. Schuetz, and Z. Stanga, "Nutritional Risk Screening and Assessment," (in eng), *J Clin Med,* vol. 8, no. 7, Jul 20 2019, doi: 10.3390/jcm8071065.

[6]	*Diagnostic and statistical manual of mental disorders: DSM-5™, 5th ed* (Diagnostic and statistical manual of mental disorders: DSM-5™, 5th ed.). Arlington, VA, US: American Psychiatric Publishing, Inc., 2013, pp. xliv, 947-xliv, 947.

[7]	P. D. E. C. S. P. Tadi, *Major Neurocognitive Disorder (Dementia)*. StatPearls: StatPearls Publishing, 2022.

[8]	S. Juma, M. M. Taabazuing, and M. Montero-Odasso, "Clinical Frailty Scale in an Acute Medicine Unit: a Simple Tool That Predicts Length of Stay," (in eng), *Can Geriatr J,* vol. 19, no. 2, pp. 34-9, Jun 2016, doi: 10.5770/cgj.19.196.

[9]	L. P. Fried *et al.*, "Frailty in older adults: evidence for a phenotype," (in eng), *J Gerontol A Biol Sci Med Sci,* vol. 56, no. 3, pp. M146-56, Mar 2001, doi: 10.1093/gerona/56.3.m146.

[10]	D. L. Murman, "The Impact of Age on Cognition," (in eng), *Semin Hear,* vol. 36, no. 3, pp. 111-21, Aug 2015, doi: 10.1055/s-0035-1555115.

[11]	A. Levin and P. E. Stevens, "Summary of KDIGO 2012 CKD Guideline: behind the scenes, need for guidance, and a framework for moving forward," (in eng), *Kidney Int,* vol. 85, no. 1, pp. 49-61, Jan 2014, doi: 10.1038/ki.2013.444.

[12]	R. C. MacCallum, S. Zhang, K. J. Preacher, and D. D. Rucker, "On the practice of dichotomization of quantitative variables," (in eng), *Psychol Methods,* vol. 7, no. 1, pp. 19-40, Mar 2002, doi: 10.1037/1082-989x.7.1.19.
